# Intratracheal obstruction caused by *Haemophilus influenzae* infection in young Children: Case report and literature review

**DOI:** 10.1097/MD.0000000000047282

**Published:** 2026-01-16

**Authors:** Yong Wu, Fu Liu, Dan Ren, Lin Yang, Yi Wu

**Affiliations:** a Department of Respiratory Medicine, Children’s Medical Center, Mianyang Central Hospital, School of Medicine, University of Electronic Science and Technology of China, Mianyang, Sichuan Province, China.

**Keywords:** flexible bronchoscopy, foreign body, *Haemophilus influenzae*, Young Children

## Abstract

**Rationale:**

According to current guidelines, rigid bronchoscopy is considered the gold standard for foreign body removal. However, we report the first case of successful removal of endobronchial necrotic material causing airway obstruction, which was associated with *Haemophilus influenzae* infection, using flexible bronchoscopy.

**Patient concerns:**

A young child presented with an airway obstruction secondary to H. influenzae infection, and culture of the viscous tracheal mass identified H. influenzae as the causative pathogen.

**Diagnoses:**

Bronchoscopic and pathological examinations further confirmed that the mass consisted of necrotic airway tissue.

**Interventions:**

Flexible bronchoscopy with irrigation and suction was performed to remove the foreign body located at the carina, effectively relieving the obstruction.

**Outcomes:**

This case demonstrates that H. influenzae infection can cause tracheal necrosis and the formation of viscous, mass-like foreign bodies that may lead to life-threatening airway obstruction, while flexible bronchoscopy proves to be an effective and minimally invasive method for their removal.

**Lessons:**

Clinicians should therefore be aware of the potential risk of airway obstruction due to viscous secretions associated with H. influenzae infection, as flexible bronchoscopy offers significant advantages in both the management and removal of such material.

## 1. Introduction

Endotracheal foreign body aspiration is a relatively common condition causing sudden respiratory distress in children. Asphyxia, coughing, and acute dyspnea are the predominant clinical manifestations.^[1]^ The age distribution of patients exhibits a bimodal pattern, primarily comprising toddlers aged 1 to 3 years and elderly individuals over 60 years old. In children, foreign body aspirations commonly involve toys and food items, whereas in the elderly population, such cases predominantly involve food.^[2]^ *Haemophilus influenzae*, a Gram-negative coccobacillus, may cause acute septic laryngitis in children.^[3]^ In severe cases, the Shwartzman reaction may occur, leading to tracheal endothelial necrosis and consequent luminal obstruction.^[4]^ The obstructive material can form a jelly-like viscous mass, which cannot be effectively removed using suction or foreign body forceps. In contrast, flexible bronchoscopy allows for simultaneous airway lavage and suction, enabling effective removal of such obstructions. This article reports a case of acute airway obstruction caused by necrotic tracheal mucosa sloughing due to *Haemophilus influenzae* infection. Flexible bronchoscopy was successfully employed as an emergency intervention to remove the obstructing material, and a review of the relevant literature is provided.

## 2. Case report

A 15-month-old girl was admitted to the hospital with a 2-day history of fever and cough, 1 day of hoarseness, and half a day of dyspnea. On admission, physical examination revealed no altered mental status, a respiratory rate of 48 breaths/min, mild inspiratory retractions, room-air oxygen saturation ranging from 92% to 94%, audible inspiratory stridor at rest, and a heart rate of 155 beats/min. Cardiovascular and abdominal examinations were unremarkable. Past medical history is unremarkable, with no family history of specific or hereditary diseases noted.

Laboratory tests revealed normal complete blood count and blood biochemistry. However, inflammatory markers were elevated: C-reactive protein (CRP)173.17 mg/L and procalcitonin (PCT) 1.64 μg/L. Mycoplasma IgM antibody was negative. Chest X-ray revealed enhanced lung texture. The initial diagnosis was acute laryngotracheitis with grade II laryngeal obstruction. Treatment was initiated with intravenous cefotaxime and methylprednisolone, along with nebulized budesonide. Four hours after treatment initiation, the patient’s dyspnea improved, and the respiratory rate decreased to 40 breaths/min.

However, 6 hours after admission, the infant became inconsolable, resisted oxygen therapy, and exhibited worsening dyspnea with cyanosis (peripheral capillary oxygen saturation (SpO_2_) dropping without oxygen support) and marked hoarseness. Immediate endotracheal intubation was performed with a 3.5 mm ID tube under parental consent, leading to significant improvement in respiratory distress and SpO_2_ stabilization at 100%.

**Figure 1.**
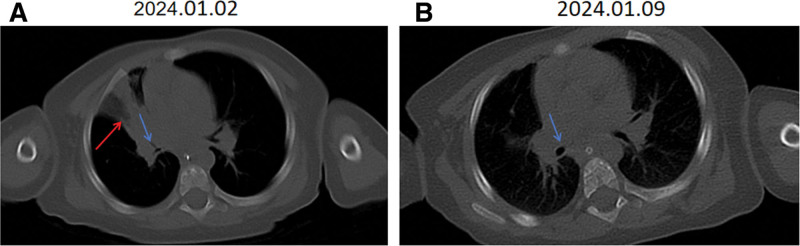
(A) The chest CT demonstrates left lung pneumonia (indicated by the red arrow) with obstructive narrowing of the left main bronchus (marked by the blue arrow). (B) One week later, a follow-up chest CT scan demonstrated complete resolution of the left lung pneumonia, with no recurrent stenosis observed in the left main bronchus (as indicated by the blue arrow). CT = computed tomography.

At 7.5 hours post-admission, while on high-parameter ventilator support, SpO_2_ remained at 92%, but bilateral lung sounds are asymmetrical, particularly in the left lung. The family denied any history of foreign body aspiration. A chest Computed tomography was performed, revealing right middle and upper lobe infiltration, consolidation, and atelectasis, suggestive of infection, along with mild bilateral lower lobe involvement. The scan also showed mucous plugs in the trachea and left main bronchus, as well as narrowing of the right intermediate and lower lobe bronchi, raising suspicion of a possible foreign body (Fig. 1A). Bedside bronchoscopy was planned.

**Figure 2.**
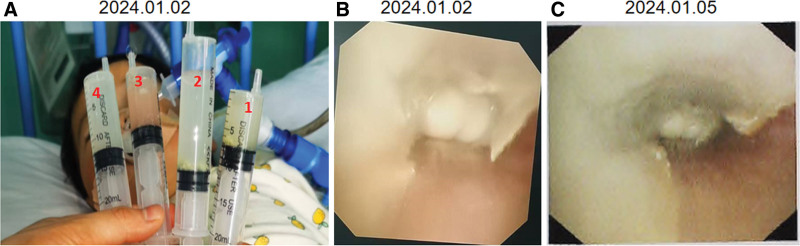
(A) Bronchoscopic lavage aspirate, with labels 1 to 4 indicating the sequential order of each lavage aspiration. Granular aspirate material is visible at the bottom of tubes 1 and 2. (B) Under bronchoscopy, abundant yellowish-white material is observed adhering to the tracheal wall and carina. (C) Three days later, repeat bronchoscopy revealed persistent necrotic debris adherent to the tracheal wall.

**Figure 3.**
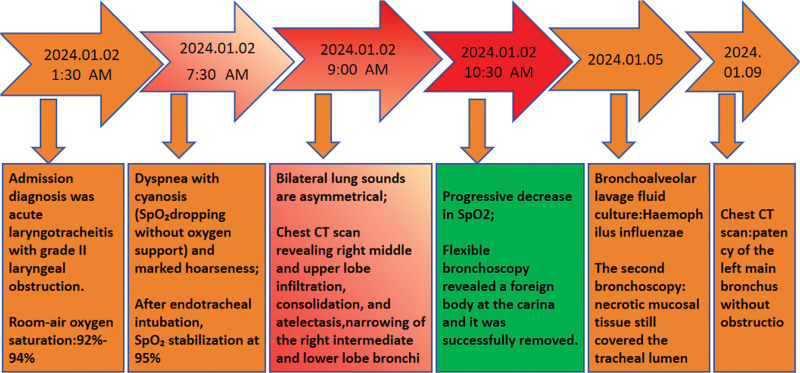
Key time points of changes in the patient’s condition after hospital admission.

By 9 hours post-admission, the child exhibited persistent desaturation in transcutaneous oxygen saturation.. Bag-valve-mask ventilation revealed increased airway resistance. The endotracheal tube was removed under anesthesia assistance, revealing thick greenish secretions obstructing the lumen. A 4.0 mm internal diameter tube was inserted under videolaryngoscopy, but SpO_2_ remained unstable. Suctioning was attempted but met resistance, suggesting distal obstruction. The child subsequently deteriorated (SpO_2_0%, heart rate 50 bpm), requiring epinephrine (Heart rate improved to 120 bpm). Due to persistent airway obstruction, the tracheal tube was removed while a flexible bronchoscope was inserted through the nasal passage. A gelatinous white mass was visualized at the carina, completely occluding the lumen. Lavage extracted copious yellowish-white granular material (Fig. 2A and B), followed by improved visualization of severely cyanosis. Post-procedure reintubation restored SpO₂to 100% and heart rate to 140 bpm. Then, the bronchoalveolar lavage fluid was collected for culture, and the lavaged particulate matter was subjected to pathological examination. The timeline of major clinical changes and resuscitation efforts following the patient’s admission is illustrated in Figure 3.

Microbiological findings: acid-fast stain of bronchoalveolar lavage fluid: Negative.

**Figure 4.**
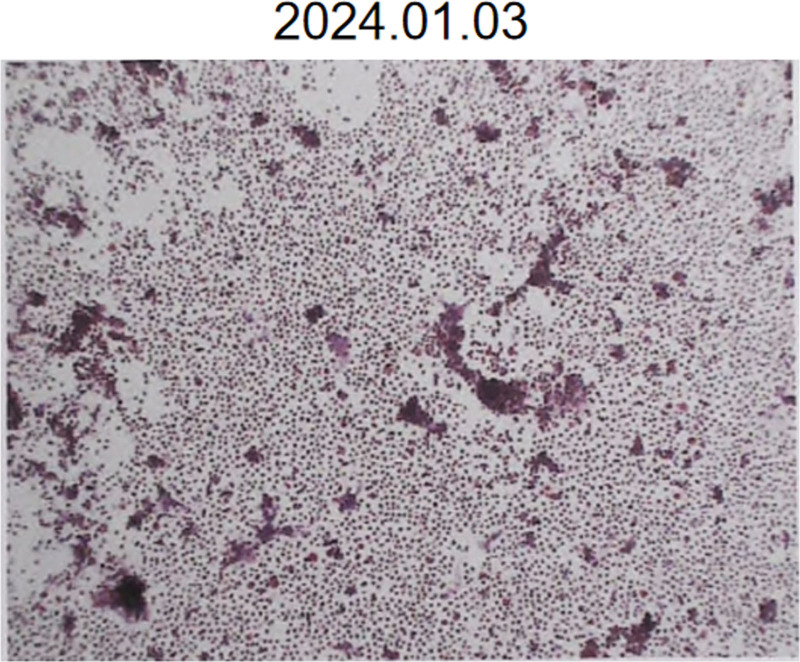
Pathological examination of the granular material revealed abundant neutrophils and scattered lymphocytes.

Pathological examination (1 day later): Abundant neutrophils with scattered lymphocytes (Fig. 4). Bronchoalveolar lavage fluid culture (3 days later): *Haemophilus influenzae* identified.

A follow-up bronchoscopy performed 3 days later revealed that necrotic mucosal tissue still covered the tracheal lumen within the main bronchus (Fig. 2C). One week later, a repeat chest computed tomography scan demonstrated resolution of pulmonary inflammation and patency of the left main bronchus without obstruction (Fig. 1B). The patient showed clinical improvement and was subsequently discharged. A 1-month telephone follow-up revealed that the child reported no specific discomfort, as relayed by the family.

## 3. Discussion

*Haemophilus influenzae* is a Gram-negative, pleomorphic coccobacillus that can be classified into 6 serotypes, with type b being the most common cause of invasive disease, followed by types d and e.^[5]^ Early animal studies have demonstrated that H. influenzae can induce mucosal damage by causing tracheal epithelial cell desquamation and impairing ciliary motility, primarily mediated by bacterial lipopolysaccharide (LPS).^[6–8]^ LPS can trigger a Shwartzman-like reaction, leading to tracheal endothelial necrosis.^[3]^

In this case report, flexible bronchoscopy revealed rapid progression to tracheal mucosal necrosis within 2 days of infection (Fig. 2A and B), further corroborating previous animal experimental findings. R. Porta Ribera et al also reported a case of H. influenzae-induced bronchitis requiring endotracheal intubation, fortunately without airway obstruction by secretions.^[9]^ However, in our case, extensive desquamated necrotic epithelial cells, impaired ciliary clearance, and diminished cough reflex due to sedation led to the formation of viscous mucus-necrotic tissue plugs obstructing the carina. Conventional suctioning failed to resolve the obstruction, resulting in life-threatening airway blockage. Performing bronchoscopy on a patient with unstable vital signs posed a significant challenge for our team. Fortunately, with the family’s support, we proceeded with the procedure. Timely intervention using flexible bronchoscopy for lavage and removal of the obstructing material ultimately saved the child’s life.

Currently, rigid bronchoscopy remains the gold standard for the diagnosis and intervention of airway foreign bodies.^[10,11]^ However, over the past decade, an increasing number of scholars have recommended the use of flexible bronchoscopy for foreign body removal, with conversion to rigid bronchoscopy when necessary.^[12]^ Flexible bronchoscopy offers advantages such as superior visualization, minimal occupation of airway space, and the ability to perform procedures under direct vision, resulting in high precision and reduced tissue damage to patients.^[13–15]^ Meanwhile, flexible bronchoscopy can also reduce surgical risks and shorten the length of hospital stay.^[16]^ In this intervention, the flexible bronchoscope effectively improved the patient’s oxygen saturation by promptly irrigating viscous secretions and aspirating necrotic tissue, demonstrating its advantage in managing viscous foreign bodies.

PCT is a crucial biomarker for diagnosing bacterial infections and sepsis.^[17]^ It can be activated by microbial toxins, interleukin-1, interleukin-6, and tumor necrosis factor-alpha.^[18]^ When the body is infected by bacteria, serum PCT levels increase significantly, with higher concentrations correlating with more severe bacterial infections.^[19]^ CRP is a nonspecific acute-phase protein synthesized by hepatocytes. As an inflammatory marker, it is easily measurable.^[20]^ The combination of white blood cell count with both PCT and CRP enhances the diagnostic accuracy for bacterial infections.^[21]^

Furthermore, patients with Gram-negative bacterial infections exhibit higher levels of PCT and CRP compared to those with Gram-positive bacterial or fungal infections.^[22]^ In the present study, following *Haemophilus influenzae* infection, the white blood cell count remained entirely normal, whereas CRP and PCT levels were markedly elevated. This observation suggests that PCT and CRP demonstrate superior sensitivity in Gram-negative bacterial infections. Moreover, a significant elevation in these markers may indicate a risk of necrotizing tracheitis.

### 3.1. The strengths and limitations in the management of this case

The strength of this case lies in the timely switch to flexible bronchoscopic lavage and suction for the removal of the airway foreign body when suctioning via the catheter proved ineffective, which successfully alleviated the patient’s respiratory distress. The limitation is that an airway assessment was not performed promptly after the initial endotracheal intubation, primarily due to the rapid deterioration of the patient’s condition shortly after admission.

## 4. Conclusion

This case highlights acute airway obstruction caused by necrotic tissue and mucus impaction in a pediatric patient with severe laryngotracheitis, necessitating urgent flexible bronchoscopic intervention. It also underscores the importance of thorough airway assessment and prompt clearance during endotracheal intubation.

## Acknowledgments

The authors sincerely appreciate the assistance of Min Liao from the Medical Records Department and Professor Gang Xie from the Pathology Department at Mianyang Central Hospital.

## Author contributions

**Conceptualization:** Yong Wu.

**Data curation:** Yong Wu, Lin Yang, Yi Wu.

**Formal analysis:** Yong Wu, Fu Liu, Dan Ren.

**Investigation:** Yong Wu, Fu Liu, Dan Ren, Lin Yang, Yi Wu.

**Methodology:** Yong Wu, Fu Liu, Yi Wu.

**Resources:** Yong Wu, Fu Liu.

**Software:** Lin Yang.

**Supervision:** Yong Wu, Dan Ren.

**Validation:** Yong Wu.

**Visualization:** Yong Wu.

**Writing – original draft:** Yong Wu.

**Writing – review & editing:** Yong Wu.
